# Organoid Models of Glioblastoma to Study Brain Tumor Stem Cells

**DOI:** 10.3389/fcell.2020.00220

**Published:** 2020-04-16

**Authors:** Roberta Azzarelli

**Affiliations:** Unit of Cell and Developmental Biology, Department of Biology, University of Pisa, Pisa, Italy

**Keywords:** glioblastoma, brain tumors, organoids, 3D models, cancer stem cells, neural stem cells, neurogenesis

## Abstract

Glioblastoma represents an aggressive form of brain cancer characterized by poor prognosis and a 5-year survival rate of only 3–7%. Despite remarkable advances in brain tumor research in the past decades, very little has changed for patients, due in part to the recurrent nature of the disease and to the lack of suitable models to perform genotype-phenotype association studies and personalized drug screening. *In vitro* culture of cancer cells derived from patient biopsies has been fundamental in understanding tumor biology and for testing the effect of various drugs. These cultures emphasize the role of *in vitro* cancer stem cells (CSCs), which fuel tumor growth and are thought to be the cause of relapse after treatment. However, it has become clear over the years that a 2D monolayer culture of these CSCs has certain disadvantages, including the lack of heterogeneous cell-cell and cell-environment interactions, which can now be partially overcome by the introduction of 3D organoid cultures. This is a novel and expanding field of research and in this review, I describe the emerging 3D models of glioblastoma. I also discuss their potential to advance our knowledge of tumor biology and CSC heterogeneity, while debating their current limitations.

## Introduction

The idea that tumor initiation, progression and regrowth after treatment are sustained by a subpopulation of cancer cells, the glioblastoma stem cells (GSCs), has been crucial to our current understating of glioblastoma (GBM) biology (Swartling et al., 2015; Alcantara Llaguno et al., 2016; Azzarelli et al., 2018; Hakes and Brand, 2019; Lu et al., 2019). Glioblastoma is a highly aggressive brain tumor characterized by elevated intratumor heterogeneity, which could be potentially attributed to variations in GSC behavior and stochastic consequences of their hierarchical growth pattern. Recent studies provided evidence for a proliferative hierarchy in GBM, by using a combination of experimental approaches, such as quantitative lineage tracing, clonal size dependences, mutational signature analysis, and single cell RNA sequencing (Patel et al., 2014; Tirosh et al., 2016; Lan et al., 2017; Neftel et al., 2019). Not only do these works indicated that tumor expansion follows a hierarchical lineage progression, but they also demonstrated that tumor cell fate decisions are rooted in a developmental program of neurogenesis. As such, early tumorigenesis is not primarily driven by genetic evolution, although genetic variations could still modulate the patterns self-renewal and differentiation of tumor cells, especially during later stages of disease progression. Evidence in another brain tumor originating in the cerebellum also showed that targeting the stem cell at the apex of a conserved developmental hierarchy could block tumor regrowth (Vanner et al., 2014). Thus, the cancer stem cell (CSC) model applied to GBM provided a framework to understand tumor heterogeneity, predict tumor evolution, and might contribute to the identification of novel therapeutic targets aimed at eliminating the GSC in order to eradicate the tumor.

**FIGURE 1:**
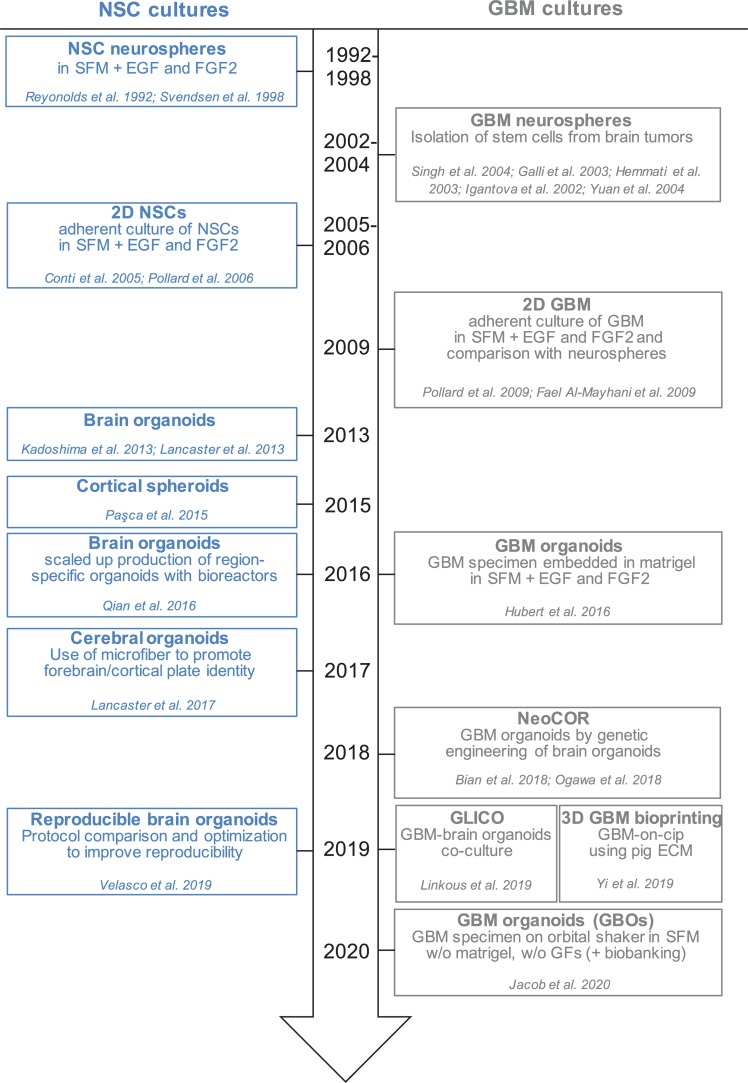
Timeline of *in vitro* method to culture neural stem cells and glioblastoma cells in 2D and 3D. Schematic representation of the development of different protocols to culture NSCs (neural stem cells) and GBM (glioblastoma) cells in monolayer, spheres and organoids. SFM, serum free medium; EGF, epidermal growth factor; FGF2, fibroblast growth factor 2; GFs, growth factors; NeoCOR, neoplastic cerebral organoids; GLICO, GLIoma cerebral organoids.

The simultaneous presence of different stem, progenitor, and differentiated cells along the developmental hierarchy and the high degree of intra-tumor heterogeneity render *in vitro* modeling of GBM particularly challenging. GSCs have been isolated from primary tumor biopsies and have been show to recapitulate *in vivo* tumor heterogeneity when forced to differentiate in culture or upon xenotransplantation (Galli et al., 2004; Singh et al., 2004; Pollard et al., 2009; Figure 1). However, when GSCs are grown in adherent 2D monolayer cultures, they lack intrinsic heterogeneity and 3D relative spatial organization, and lose interactions with the diverse components of the tumor extracellular matrix and the microenvironment. Moreover, these cells scarcely predict treatment efficacy, as drugs that initially proved effective in the context of cultured cell lines did not result in clinical applications (Zanders et al., 2019).

Thus, more refined model systems that allow the recapitulation of complex cancer phenotypes and yet retain the amenability to perform detailed analysis are needed, especially in view of the need to provide more accurate predictions of the therapeutic potential of new treatments. Encouraged by promising results in other cancer fields (Boj et al., 2015; Van De Wetering et al., 2015; Sachs et al., 2018; Tuveson and Clevers, 2019), several laboratories have directed their efforts to generate organoid models of glioblastoma, which consist, by definition, of 3D structures in which different cell types self-organize to establish appropriate cell–cell contacts and to create a microenvironment (Huch and Koo, 2015). As such, GBM organoids could better mimic tumor complexity and heterogeneity in growth potential and treatment responsiveness. This review describes the existing organoid GBM models that have just started to be developed and compares them to other 3D models, such as neurospheres and 3D bioprinted GBMs. It also discusses their potential to advance our understanding of GBM biology and to predict clinical outcome, while also considering their current limitations.

## Three-Dimensional Models of Glioblastoma

### Tumorspheres and Glioblastoma Organoids From Primary Tissue

Glioblastoma stem cells can be isolated from primary tumors and can be grown in culture for an extended period of time (Ignatova et al., 2002; Hemmati et al., 2003; Galli et al., 2004; Singh et al., 2004; Tunici et al., 2004; Yuan et al., 2004; Fael Al-Mayhani et al., 2009; Pollard et al., 2009; Vukicevic et al., 2010; Figure 1 and Table 1). *In vitro* expansion of GSC is sustained by growth factors like EGF (Epidermal Growth Factor) and FGF2 (Fibroblast Growth Factor), conditions that also expand neural stem cells, highlighting the close relationship between GSC and their normal counterpart (Conti et al., 2005; Pollard et al., 2006, 2009). GSC can be grown in 2D adherent culture or as 3D neurospheres: the latter can be considered the very first “3D model” of GBM, as cells maintain a certain degree of polarization and 3D spatial organization (Galli et al., 2004; Azari et al., 2011). However, neurospheres are characterized by a necrotic core and can thus be able to achieve a maximum size of around 300 μm, before needing disruption and replating to survive (Reynolds and Weiss, 1992; Svendsen et al., 1998; Reynolds and Rietze, 2005). In addition, cells in neurospheres have lost their interaction with components of the extracellular matrix, and thus hardly mimic *in vivo* GSC behavior (Table1).

**TABLE 1:** Overview of the characteristics of the different methods.

	**2D**	**Spheres**	**Organoids**			
			**No matrigel GBO**	**Matrigel**	**Genetic Eng. NeoCOR**	**Co-culture GLICO**
**Efficiency of derivation**	100%	<50%	91.4%	n.d.	Oncogene-dependent	100%

**Time of culture establishment**	1–2 weeks	1–2 weeks	1–2 weeks	1–2 months	1–4 months	1–2 months

**Homogeneity (bulk analysis)**	+	±	–	–	–	–

**Heterogeneity (maintenance of tumor complexity)**	–	–	+	+	+	+

**Relative 3D spatial distribution**	–	–	+	+	+	+

**Genetic stability overtime**	±^a^	±	+^b^	n.d^c^	n.a.^d^	+

**Freeze/thaw**	+	+	+	–	–	–

**Maximum time in culture**	>1 year	6–9 months	>1 month	>1 year	1–2 months post electroporation	14–24 days post co-culture

**Potential to predict response to treatment**	–	–	+	n.d.	+	+

**GBM/non-GBM cell mix to study invasion**	–	–	–	–	+	+

**References**	Fael Al-Mayhani et al., 2009; Pollard et al., 2009	Galli et al., 2004; Tunici et al., 2004; Pollard et al., 2009; Vukicevic et al., 2010	Jacob et al., 2020	Hubert et al., 2016	Bian et al., 2018; Ogawa et al., 2018	Ogawa et al., 2018; Linkous et al., 2019

*^*a*^Genomic stability is maintained within the first 6 months of culture (Pollard et al., 2009); some features are rapidly lost, such as EGFR amplification (Fael Al-Mayhani et al., 2009; Linkous et al., 2019). ^*b*^Genomic stability is maintained, but the analysis has been done at only 2 weeks in culture (Jacob et al., 2020). ^*c*^n.d. not determined. ^*d*^n.a., not applicable. GBO, GBM organoid; NeoCOR, neoplastic cerebral organoids; GLICO, GLIoma cerebral organoids.*

**FIGURE 2:**
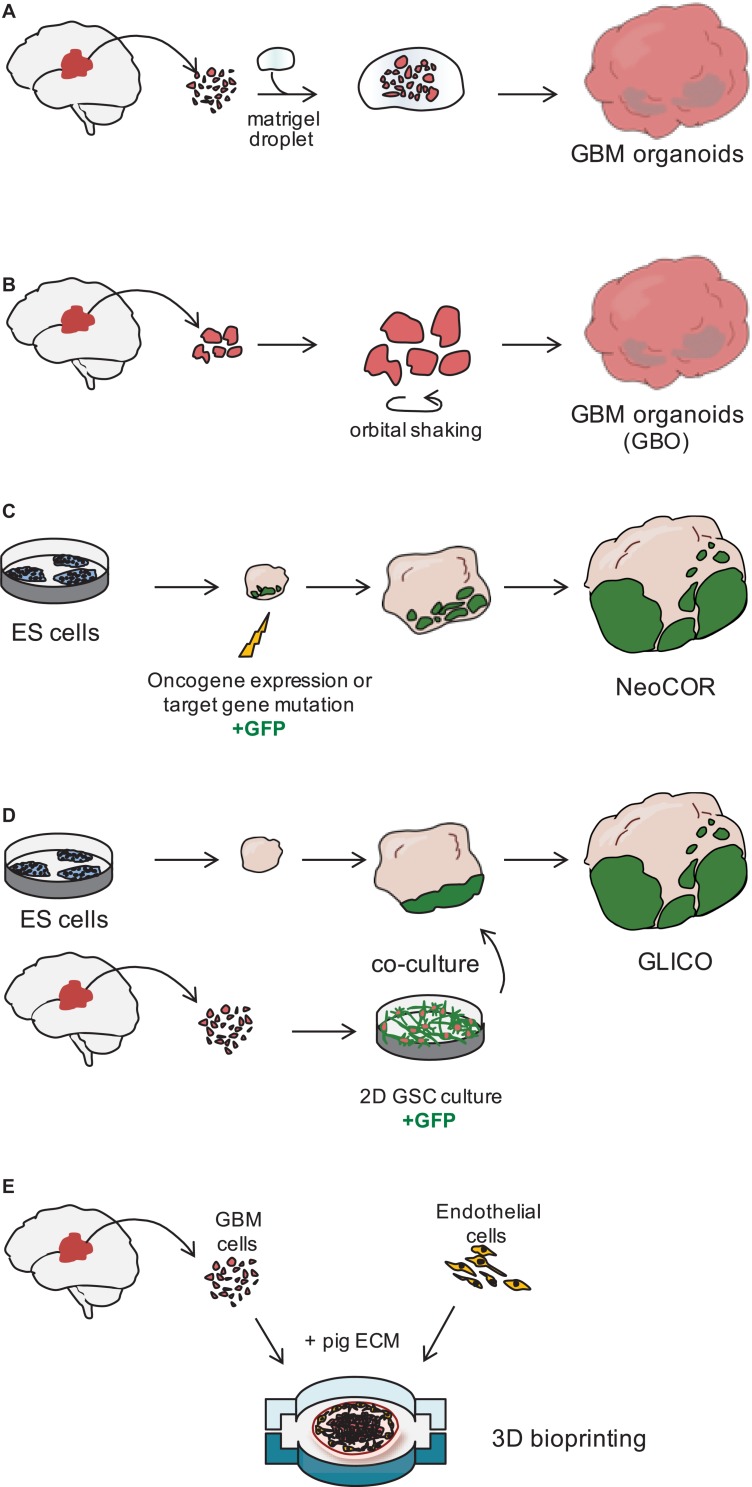
Three dimensional models of glioblastoma. **(A)** Glioblastoma (GBM) organoids have been derived by embedding finely minced GBM specimen in matrigel (Hubert et al., 2016) or **(B)** by culturing pieces of tumor biopsies in defined matrigel-free and serum-free conditions, on an orbital shaker. GBO. GBM Organoid (Jacob et al., 2020). **(C)** Embryonic stem (ES) cell-derived brain organoids can be nucleofected at early stages of the differentiation to introduce tumor-promoting genetic alterations. During nucleofection, cells are also marked with green fluorescent protein (GFP) to visualize tumor cell growth. NeoCOR, neoplastic cerebral organoids (Bian et al., 2018; Ogawa et al., 2018). **(D)** Patient-derived glioblastoma stem cells (GSCs) are initially cultured in 2D, before being co-cultured with brain organoids. GSCs are marked by GFP to visualize integration and growth in the organoid. GLICO, GLIoma cerebral organoids (Ogawa et al., 2018; Linkous et al., 2019). **(E)** Patient-derived GBM cells and endothelial cells are seeded on a chip using a pig extracellular matrix (ECM) bio-ink and a 3D bioprinter (Yi et al., 2019).

In 2016, the lab of Jeremy Rich developed *in vitro* conditions to grow 3D organoids from human GBM cells and from GBM biopsies. When embedded in matrigel, finely minced GBM specimens grew up to 3–4 mm in 2 months and could be kept in culture for over a year (Table 1), even if their growth slows down after several months, probably due to the limited diffusion of nutrients as the organoids grow in size (Hubert et al., 2016; Figure 2A). An interesting feature of these GBM organoids is that they recapitulate the gradient of stem cell density in relation to hypoxic levels found *in vivo*. The authors reported a high number of Sox2+ stem cells at the periphery of the organoid, while the core was characterized by lower abundance of Sox2+ cells and increased levels of hypoxia. Sox2+ stem cells also exhibited different molecular properties when located at the core or at the periphery of the organoids (Hubert et al., 2016). Thus, multiple Sox2+ populations may co-exist in organoids, suggesting that the organoid microenvironment might be able to sustain the simultaneous growth of different CSCs and would allow the study of cellular hierarchies in tumors. While highly promising, this system would require further characterization and validation across several GBMs. Indeed, establishment rates have yet to be determined and may be very patient-specific. Moreover, this system suffers from a relatively low to medium throughput capability and the long time necessary to establish the cultures (1–2 months) (Table 1).

Recently, a novel and faster protocol (1–2 weeks) of 3D GBM organoid derivation that overcome such limitations has been reported by collaboration between Donald O’Rourke, Guo-li Ming, and Hongjun Song at the University of Pennsylvania (Table 1 and Figure 1). Instead of dissociating tumor biopsies to fine pieces, the authors cut the biopsies into around 1 mm fragments and culture them on an orbital shaker without matrigel and in serum free conditions not supplemented with EGF and FGF2 (Figure 2B; Jacob et al., 2020). These conditions should avoid selection of specific cell populations, thus better preserving inter- and intra-tumoral heterogeneity. These glioblastoma organoids, called GBOs, also developed hypoxic gradient and were further propagated in culture by cutting them into smaller pieces to avoid inner core necrosis. Importantly, these GBOs were cryopreserved and able to recover and continue their growth upon thawing. This is an essential step to generate GBM biobanks for subsequent recovery and analysis and the authors have currently biobanked around 70 GBOs from 53 patients (sometimes including different tumor subregions). GBOs largely maintain genetic and molecular signatures of the parental tumors. However, most analyses have been done within the first two weeks in culture, as GBOs maintenance over very long periods has been variable.

This novel protocol is fast and reproducible and provides enough material for RNA and exome sequencing, as well as drug sensitivity tests. It is thus suitable for genotype-drug association studies and opens new avenues to personalized medicine approaches, along the line of current advances in other cancer fields in which organoid biobanks have already been established or are currently being generated (Boj et al., 2015; Van De Wetering et al., 2015; Sachs et al., 2018; Yan et al., 2018; Kim et al., 2019).

The establishment of the cultures within 1–2 weeks from surgical resection is particularly important, because current treatments are initiated within a month post-surgery and having preclinical information about potentially effective treatments might be extremely useful and might also help refine patient enrolment in clinical trials. In this direction, GBO treatment with CAR-T cells against EGFRvIII variant, which is currently in clinical trials (O’Rourke et al., 2017; Goff et al., 2019), resulted in specific effect only on GBOs containing high percentage of EGFRvIII+ cells, thus showing the translational impact and future pre-clinical potential of this approach (Jacob et al., 2020).

### Tumor Development by Genetic Engineering of Brain Organoids

The development of human brain organoids or “minibrians” have revolutionized the way we operate in developmental neurobiology, by providing unprecedented access to aspects of human brain development functioning and disorders (Kadoshima et al., 2013; Lancaster et al., 2013, 2017; Paşca et al., 2015; Qian et al., 2016, 2018, 2019; Amin and Paşca, 2018; Figure 1). The potential of brain organoids to recapitulate aspects of brain cancer is particularly valuable as neither patient derived xenotransplantation in mice nor human brain tumor stem cells in 2D culture behave in the same way as tumors *in vivo*.

The labs of Jürgen Knoblich and Inder Verma have recently genetically engineered organoids to develop tumors (Bian et al., 2018; Ogawa et al., 2018; Figures 1, 2C and Table 1). Bian et al. (2018) screened for genetic alterations that could lead to tumorigenesis and called the resulting tumor NeoCor (neoplastic cerebral organoids): the authors overexpressed known oncogenes by a transposase-based system and/or deleted tumor suppressor gene functions via CRIPSR Cas-9. Organoid cells have been targeted by nucleofection at very early stages of the differentiation and the cells carrying the genetic alterations have been marked by GFP, so that cell growth and tumor transformation could be easily followed. This screening identified MYC overexpression and a few more genetic combinations often found in human GBM to provide cells with a strong growth advantage. Transcriptomic profiling showed that MYC overexpressing tumors have a CNS-PNET-like identity (CNS-PNET: Primitive Neuro-Ectodermal Tumor of the Central Nervous System), while the other tumors resemble GBM more, suggesting that distinct genetic aberrations can induce tumors with specific cellular identities. Other works have also shown that mesenchymal GBM can be induced in organoids by defined genetic mutations, namely HRasG12V activation and p53 disruption (Ogawa et al., 2018).

Although these works show that a certain degree of GBM subtyping can be reproduced in organoids, whether all different GBM subtypes can be recapitulated in this system and how closely GBM-derived organoids resemble patient-derived GBM cells is still under investigated. Tumors other than GBM did not develop despite the genetic manipulation of genes classically altered in these tumors, such as the deletion of the inhibitory Sonic Hedgehog (SHH) receptor PTCH1 in SHH-group medulloblastoma (Bian et al., 2018). However, the oncogenic effect of PTCH1 deletion is known to be cell-type specific (Schüller et al., 2008) and might therefore necessitate organoid pre-differentiation down the cerebellar route for transformation to occur (Ballabio et al., 2020).

### Co-culture of GBM Cells With Brain Organoids

Tumor models created by genetic engineering of organoids described above are particularly advantageous to effectively model GBM initiation, but they hardly recapitulate the genomic complexity of *in vivo* tumors, as the methodology requires genetic manipulation of the few known driver genes, which are not necessarily representative of the genomic GBM heterogeneity. The laboratory of Howard Fine and other laboratories have recently developed a novel approach that overcome this disadvantage, by co-culturing patient-derived GSCs with 3D brain organoids and called their model GLICO (GLIoma cerebral organoids; Linkous et al., 2019; Figures 1, 2D and Table 1). The authors co-cultured different GFP-marked GSC cell lines with fully grown cerebral brain organoids and demonstrated that GSCs proliferate over time and integrate into the organoids. Each line behaves in a unique way, with some lines showing diffuse invasion, others forming “honeycomb”-like structures and others forming small regional nodes of proliferation (Linkous et al., 2019). Interestingly, co-cultured GSCs that exhibit higher degree of invasiveness were also more lethal when transplanted in mice (Ogawa et al., 2018). Thus, the observed heterogeneity in growth and invasion in the GLICO model likely reflects certain intrinsic properties of that particular patient-derived GSC line.

Cancer cells in this system not only displayed a cellular behavior that closely mimics the original tumor, but they also maintained key genetic aberrations of the native tumor. EGFR amplification, which was identified in two of the analyzed cell lines, was maintained in the GLICO models, while often lost in 2D cultures (Linkous et al., 2019; Table 1), indicating that this model may provide a more suitable microenvironment to preserve the genetic background of the *in vivo* tumor.

### Three-Dimensional GBM Model via Bioprinting

The use of advanced 3D bioprinting technologies could enhance the way we design 3D GBM models *in vitro* (van Pel et al., 2018; Yi et al., 2019). Yi et al. (2019) created a GBM-on-chip model, in which they used decellularized pig brain extracellular matrix as a bio-ink to seed patient-derived cancer cells together with vascular endothelial cells in separated compartments (Figure 2E). Compartmentalization by seeding endothelial cells on the outside and cancer cells in the core of the chip established a radial oxygen gradient, which recapitulated important pathological features of the tumor. This model indeed exhibited hypoxia induced necrotic core, a perivascular niche and maintained a degree of spatial heterogeneity of the different cell types, with the higher number of Sox2+ stem cells at the periphery of the seeded tumor.

The “biomimetic” conditions of this system provided a microenvironment comparable to that of the original *in vivo* tumor tissue, offering the advantage of promoting cell-cell and cell-matrix interactions, and of better predicting treatment responses in a shorter time frame than other models (1–2 weeks compared to 1–2 months). However, the system still lacks accurate 3D spatial organization that can be only generated using self-assembled 3D organoid cultures, and it requires advanced technologies and expertise not always available to common biological laboratories.

## Advantages and Limitations: Which Model to Use?

The best GBM model would be one that is complex enough to recapitulate features of the original tumor and simple enough to support investigation of different aspects of carcinogenesis in isolation. While the focus of this review is to look at emerging 3D *in vitro* models of GBM, several other approaches, including engineered mouse models or xenotransplantation, have been particularly useful to address tumor biology in other contexts [for a review of GBM models *in vivo* and *in vitro*, see Robertson et al. (2019)]. Thus, researchers might have to balance pros and cons of the different models to find the best fit for their research question, and might have to combine more than one model to take advantage of their complementary strengths (Table 1).

The development of 3D *in vitro* models of GBM holds great potential to study GBM biology and predict response to treatment, as they more closely recapitulate the complexity and heterogeneity of the original tumor. Indeed, most of the 3D organoid models described here have also shown selective vulnerabilities for targeted therapies or radiation that closely resemble tumor sensitivity *in vivo* (Hubert et al., 2016; Bian et al., 2018; Linkous et al., 2019). In addition, the recent establishment of 3D GBM organoids from biopsies with a novel and faster protocol (1–2 weeks) promoted the generation of a live GBM biobank that can be used for genotype-drug association studies on a medium to high throughput capability (Jacob et al., 2020). Thus, 3D GBM models provide a powerful predicting tool that could be used one day to guide clinical decisions.

Some of the models described in this review (Bian et al., 2018; Ogawa et al., 2018; Linkous et al., 2019) and other similar models (da Silva et al., 2018; Plummer et al., 2019) allow the possibility to mix GBM and non-GBM brain cells (Table 1). This is particularly useful to study tumor invasion of the normal tissue and the interaction of tumor cells with normal brain cells. By targeting only one or the other compartment at a time, it will thus be possible to dissect the specific role of genes involved in cell-cell interaction, adhesion, guidance and migration, and this might identify novel therapeutical targets to block tumor infiltration.

The brain organoid tissue GBM cells interact with, however, resembles more an embryonic type of tissue, rather than the adult brain tissue of GBM derivation. At present, it is not clear how this might influence tumor properties. In the future, it would be interesting to develop a similar approach to model and study prenatal and childhood tumors, such as pediatric gliomas and medulloblastomas, which should maintain a closer link to their developmental origin (Liu and Zong, 2012; Azzarelli et al., 2018; Lu et al., 2019). As medulloblastoma did not develop in organoids, even when genetic alterations typical of this tumors were introduced, it might be necessary to generate regionalized organoids (Muguruma et al., 2015; Dias and Guillemot, 2017; Renner et al., 2017; Ballabio et al., 2020) tailored to the area of origin of that specific tumor, prior to transformation.

An aspect that has likely benefited from having this embryonic type of tissue, is the maintenance of the CSC compartment of the tumor. By providing cancer cells with the more appropriate environment that could support the simultaneous presence of different stem and progenitor cells, these 3D GBM organoid models will foster the investigation of CSC heterogeneity (Bhaduri et al., 2020). They will also open the possibility to study CSC developmental hierarchy in tumors and the influence of other cell types or of the environment on CSC fate decisions. While it is possible to incorporate non-neuronal cell types into organoids, such as microglia or other immune cells (Abud et al., 2017; Brownjohn et al., 2018; Ormel et al., 2018; Jacob et al., 2020), the main challenge still remains to recreate an environment that includes the vasculature and other cell types that could exhibit inflammatory and immunitary responses similar to an intact brain (Daviaud et al., 2018; Lancaster, 2018; Mansour et al., 2018).

The presence of different cell types and the high degree of heterogeneity is probably the main advantage and, at the same time, a disadvantage of the system because, while it reflects the complexity of the original tumor, it is probably the source of variability typical of these 3D cultures (Lancaster and Knoblich, 2014; Quadrato et al., 2017; Amin and Paşca, 2018; Qian et al., 2019; Velasco et al., 2019; Table 1). Thus, investigators might have to choose between growing cells in classical 2D monolayer or sphere cultures or in 3D organoids depending on whether they are more interested in performing bulk analysis on an homogenous cell population or whether they aim to investigate tumor aspects that requires maintenance of tumor complexity and heterogeneity.

## Conclusion and Perspectives

The emerging development of 3D organoids of GBM adds on to an abundance of choices to model this aggressive brain tumor (Robertson et al., 2019; Figure 1 and Table 1). It provides researchers with an additional tool to understand GBM biology, and predict tumor progression and response to treatment. One of the main advantages of growing GBM in brain organoids is the possibility to simultaneously grow different stem cells, progenitors and their differentiated progeny within the same conditions (Hubert et al., 2016; Jacob et al., 2020). This not only mimics better the heterogeneity of the original tumor, but it also shows the persistence of developmental programs of neurogenesis in the tumor. By understanding how CSCs make fate decisions and by defining the aberrant developmental pathways that lead to tumorigenesis, it will be possible to exploit novel emerging vulnerabilities to kill or differentiate CSCs to eradicate the tumor.

Future challenges include the reduction of organoid variability, while maintaining tumor complexity and heterogeneity, and the incorporation of an appropriate microenvironment that could, for example, mimic inflammatory and immunological responses. This would be particularly relevant in view of current successes in cancer immunotherapy (Fesnak et al., 2016) and, once incorporated in organoids, it would help to understand how immunological response might influence CSC hierarchy, and tumor progression and regrowth (Weller et al., 2017; Lim et al., 2018).

Our knowledge on GBM has massively expanded in the past decades and future collaborations between oncologists, clinicians, and researchers in the cancer, stem cell, and developmental biology fields, together with the possibility to share different complementary models and tools are likely to bring the long-sought breakthroughs that will improve patient treatment and prognosis.

## Author Contributions

RA wrote the manuscript and prepared the figures.

## Conflict of Interest

The author declares that the research was conducted in the absence of any commercial or financial relationships that could be construed as a potential conflict of interest.

## References

[B1] Abud E. M. Ramirez R. N. Martinez E. S. Healy L. M. Nguyen C. H. H. Newman S. A. (2017). iPSC-derived human microglia-like cells to study neurological diseases. *Neuron* 94 278–293.e9. 10.1016/j.neuron.2017.03.042. 28426964 PMC5482419

[B2] Alcantara Llaguno S. R. Xie X. Parada L. F. (2016). Cell of origin and cancer stem cells in tumor suppressor mouse models of glioblastoma. *Cold Spring Harb. Symp. Quant. Biol.* 81 31–36. 10.1101/sqb.2016.81.030973 27815542 PMC6353557

[B3] Amin N. D. Paşca S. P. (2018). Building models of brain disorders with three-dimensional organoids. *Neuron* 100 389–405. 10.1016/j.neuron.2018.10.007 30359604

[B4] Azari H. Millette S. Ansari S. Rahman M. Deleyrolle L. P. Reynolds B. A. (2011). Isolation and expansion of human glioblastoma multiforme tumor cells using the neurosphere assay. *J. Vis. Exp.* 56:e3633 10.3791/3633 PMC3227195 22064695

[B5] Azzarelli R. Simons B. D. Philpott A. (2018). The developmental origin of brain tumours: a cellular and molecular framework. *Development* 145:dev162693 10.1242/dev.162693 PMC6001369 29759978

[B6] Ballabio C. Anderle M. Gianesello M. Lago C. Miele E. Cardano M. (2020). Modeling medulloblastoma in vivo and with human cerebellar organoids. *Nat. Commun.* 11 1–18. 31996670 10.1038/s41467-019-13989-3 PMC6989674

[B7] Bhaduri A. Di Lullo E. Jung D. Müller S. Crouch E. E. Espinosa C. S. (2020). Outer radial glia-like cancer stem cells contribute to heterogeneity of glioblastoma. *Cell Stem Cell* 26 48–63.e6. 10.1016/j.stem.2019.11.015 31901251 PMC7029801

[B8] Bian S. Repic M. Guo Z. Kavirayani A. Burkard T. Bagley J. A. (2018). Genetically engineered cerebral organoids model brain tumor formation. *Nat. Methods* 15 631–639. 10.1038/s41592-018-0070-7 30038414 PMC6071863

[B9] Boj S. F. Hwang C. I. Baker L. A. Chio I. I. C. Engle D. D. Corbo V. (2015). Organoid models of human and mouse ductal pancreatic cancer. *Cell* 160 324–338. 10.1016/j.cell.2014.12.021 25557080 PMC4334572

[B10] Brownjohn P. W. Smith J. Solanki R. Lohmann E. Houlden H. Hardy J. (2018). Functional studies of missense TREM2 mutations in human stem cell-derived microglia. *Stem Cell Rep.* 10 1294–1307. 10.1016/j.stemcr.2018.03.003 PMC5998752 29606617

[B11] Conti L. Pollard S. M. Gorba T. Reitano E. Toselli M. Biella G. (2005). Niche-independent symmetrical self-renewal of a mammalian tissue stem cell. *PLoS Biol.* 3:1594–1606. 10.1371/journal.pbio.0030283 PMC1184591 16086633

[B12] da Silva B. Mathew R. K. Polson E. S. Williams J. Wurdak H. (2018). Spontaneous glioblastoma spheroid infiltration of early-stage cerebral organoids models brain tumor invasion. *SLAS Discov.* 23 862–868. 10.1177/2472555218764623 29543559

[B13] Daviaud N. Friedel R. H. Zou H. (2018). Vascularization and engraftment of transplanted human cerebral organoids in mouse cortex. *eNeuro* 5 1–18. 10.1523/ENEURO.0219-18.2018 PMC6243198 30460331

[B14] Dias C. Guillemot F. (2017). Revealing the inner workings of organoids. *EMBO J.* 36 1299–1301. 10.15252/embj.201796860 28438893 PMC5430205

[B15] Fael Al-Mayhani T. M. Ball S. L. R. Zhao J. W. Fawcett J. Ichimura K. Collins P. V. (2009). An efficient method for derivation and propagation of glioblastoma cell lines that conserves the molecular profile of their original tumours. *J. Neurosci. Methods* 176 192–199. 10.1016/j.jneumeth.2008.07.022 19215724

[B16] Fesnak A. D. June C. H. Levine B. L. (2016). Engineered T cells: the promise and challenges of cancer immunotherapy. *Nat. Rev. Cancer* 16 566–581. 10.1038/nrc.2016.97 27550819 PMC5543811

[B17] Galli R. Binda E. Orfanelli U. Cipelletti B. Gritti A. De Vitis S. (2004). Isolation and characterization of tumorigenic, stem-like neural precursors from human glioblastoma (Cancer Research (October 2004) 64 (7011-7021). *Cancer Res.* 64:8130. 10.1158/0008-5472.CAN-04-1364 15466194

[B18] Goff S. L. Morgan R. A. Yang J. C. Sherry R. M. Robbins P. F. Restifo N. P. (2019). Pilot trial of adoptive transfer of chimeric antigen receptor-Transduced t cells targeting egfrviii in patients with glioblastoma. *J. Immunother.* 42 126–135. 10.1097/CJI.0000000000000260 30882547 PMC6691897

[B19] Hakes A. E. Brand A. H. (2019). Neural stem cell dynamics: the development of brain tumours. *Curr. Opin. Cell Biol.* 60 131–138. 10.1016/j.ceb.2019.06.001 31330360

[B20] Hemmati H. D. Nakano I. Lazareff J. A. Masterman-Smith M. Geschwind D. H. Bronner-Fraser M. (2003). Cancerous stem cells can arise from pediatric brain tumors. *Proc. Natl. Acad. Sci. U.S.A.* 100 15178–15183. 10.1073/pnas.2036535100 14645703 PMC299944

[B21] Hubert C. G. Rivera M. Spangler L. C. Wu Q. Mack S. C. Prager B. C. (2016). A three-dimensional organoid culture system derived from human glioblastomas recapitulates the hypoxic gradients and cancer stem cell heterogeneity of tumors found in vivo. *Cancer Res.* 76 2465–2477. 10.1158/0008-5472.can-15-2402 26896279 PMC4873351

[B22] Huch M. Koo B. K. (2015). Modeling mouse and human development using organoid cultures. *Development* 142 3113–3125. 10.1242/dev.118570 26395140

[B23] Ignatova T. N. Kukekov V. G. Laywell E. D. Suslov O. N. Vrionis F. D. Steindler D. A. (2002). Human cortical glial tumors contain neural stem-like cells expressing astroglial and neuronal markers in vitro. *Glia* 39 193–206. 10.1002/glia.10094 12203386

[B24] Jacob F. Salinas R. D. Zhang D. Y. Rourke D. M. O. (2020). Resource a patient-derived glioblastoma organoid model and resource a patient-derived glioblastoma organoid model and biobank recapitulates inter- and intra-tumoral heterogeneity. *Cell* 180 188–204.e22. 10.1016/j.cell.2019.11.036 31883794 PMC7556703

[B25] Kadoshima T. Sakaguchi H. Nakano T. Soen M. Ando S. Eiraku M. (2013). Self-organization of axial polarity, inside-out layer pattern, and species-specific progenitor dynamics in human ES cell-derived neocortex. *Proc. Natl. Acad. Sci. U.S.A.* 110 20284–20289. 10.1073/pnas.1315710110 24277810 PMC3864329

[B26] Kim M. Mun H. Sung C. O. Cho E. J. Jeon H. J. Chun S. M. (2019). Patient-derived lung cancer organoids as in vitro cancer models for therapeutic screening. *Nat. Commun.* 10:3991. 10.1038/s41467-019-11867-6 PMC6728380 31488816

[B27] Lan X. Jörg D. J. Cavalli F. M. G. Richards L. M. Nguyen L. V. Vanner R. J. (2017). Fate mapping of human glioblastoma reveals an invariant stem cell hierarchy. *Nature* 549 227–232. 10.1038/nature23666 28854171 PMC5608080

[B28] Lancaster M. A. (2018). Brain organoids get vascularized. *Nat. Biotechnol.* 36 407–408. 10.1038/nbt.4133 29734310

[B29] Lancaster M. A. Corsini N. S. Wolfinger S. Gustafson E. H. Phillips A. W. Burkard T. R. (2017). Guided self-organization and cortical plate formation in human brain organoids. *Nat. Biotechnol.* 35 659–666. 10.1038/nbt.3906 28562594 PMC5824977

[B30] Lancaster M. A. Knoblich J. A. (2014). Generation of cerebral organoids from human pluripotent stem cells. *Nat. Protoc.* 9 2329–2340. 10.1038/nprot.2014.158 25188634 PMC4160653

[B31] Lancaster M. A. Renner M. Martin C. A. Wenzel D. Bicknell L. S. Hurles M. E. (2013). Cerebral organoids model human brain development and microcephaly. *Nature* 501 373–379. 10.1038/nature12517 23995685 PMC3817409

[B32] Lim M. Xia Y. Bettegowda C. Weller M. (2018). Current state of immunotherapy for glioblastoma. *Nat. Rev. Clin. Oncol.* 15 422–442. 10.1038/s41571-018-0003-5 29643471

[B33] Linkous A. Balamatsias D. Snuderl M. Edwards L. Miyaguchi K. Milner T. (2019). Modeling patient-derived glioblastoma with cerebral organoids. *Cell Rep.* 26 3203–3211.e5. 10.1016/j.celrep.2019.02.063 30893594 PMC6625753

[B34] Liu C. Zong H. (2012). Developmental origins of brain tumors. *Curr. Opin. Neurobiol.* 22 844–849. 10.1016/j.conb.2012.04.012 22560511 PMC3432164

[B35] Lu Q. R. Qian L. Zhou X. (2019). Developmental origins and oncogenic pathways in malignant brain tumors. *Wiley Interdiscip. Rev. Dev. Biol.* 8 1–23. 10.1002/wdev.342 PMC6565468 30945456

[B36] Mansour A. A. Gonçalves J. T. Bloyd C. W. Li H. Fernandes S. Quang D. (2018). An in vivo model of functional and vascularized human brain organoids. *Nat. Biotechnol.* 36 432–441. 10.1038/nbt.4127 29658944 PMC6331203

[B37] Muguruma K. Nishiyama A. Kawakami H. Hashimoto K. Sasai Y. (2015). Self-organization of polarized cerebellar tissue in 3D culture of human pluripotent stem cells. *Cell Rep.* 10 537–550. 10.1016/j.celrep.2014.12.051 25640179

[B38] Neftel C. Laffy J. Filbin M. G. Hara T. Shore M. E. Rahme G. J. (2019). An integrative model of cellular states, plasticity, and genetics for glioblastoma. *Cell* 178 835–849.e21. 10.1016/j.cell.2019.06.024 31327527 PMC6703186

[B39] Ogawa J. Pao G. M. Shokhirev M. N. Verma I. M. (2018). Glioblastoma model using human cerebral organoids. *Cell Rep.* 23 1220–1229. 10.1016/j.celrep.2018.03.105 29694897 PMC6892608

[B40] Ormel P. R. Vieira, de Sá R. van Bodegraven E. J. Karst H. Harschnitz O. (2018). Microglia innately develop within cerebral organoids. *Nat. Commun.* 9:4167. 10.1038/s41467-018-06684-2 PMC6177485 30301888

[B41] O’Rourke D. M. Nasrallah M. P. Desai A. Melenhorst J. J. Mansfield K. Morrissette J. J. D. (2017). A single dose of peripherally infused EGFRvIII-directed CAR T cells mediates antigen loss and induces adaptive resistance in patients with recurrent glioblastoma. *Sci. Transl. Med.* 9:eaaa0984 10.1126/scitranslmed.aaa0984 PMC5762203 28724573

[B42] Paşca A. M. Sloan S. A. Clarke L. E. Tian Y. Makinson C. D. Huber N. (2015). Functional cortical neurons and astrocytes from human pluripotent stem cells in 3D culture. *Nat. Methods* 12:671 10.1038/nmeth.3415 PMC4489980 26005811

[B43] Patel A. P. Tirosh I. Trombetta J. J. Shalek A. K. Gillespie S. M. Wakimoto H. (2014). Single-cell RNA-seq highlights intratumoral heterogeneity in primary glioblastoma. *Science (80-)* 344 1396–1401. 10.1126/science.1254257 PMC4123637 24925914

[B44] Plummer S. Wallace S. Ball G. Lloyd R. Schiapparelli P. Quiñones-Hinojosa A. (2019). A Human iPSC-derived 3D platform using primary brain cancer cells to study drug development and personalized medicine. *Sci. Rep.* 9 1–11. 30723234 10.1038/s41598-018-38130-0 PMC6363784

[B45] Pollard S. M. Conti L. Sun Y. Goffredo D. Smith A. (2006). Adherent neural stem (NS) cells from fetal and adult forebrain. *Cereb. Cortex* 16 i112–i120. 10.1093/cercor/bhj167 16766697

[B46] Pollard S. M. Yoshikawa K. Clarke I. D. Danovi D. Stricker S. Russell R. (2009). Glioma stem cell lines expanded in adherent culture have tumor-specific phenotypes and are suitable for chemical and genetic screens. *Cell Stem Cell* 4 568–580. 10.1016/j.stem.2009.03.014 19497285

[B47] Qian X. Jacob F. Song M. M. Nguyen H. N. Song H. Ming G. L. (2018). Generation of human brain region–specific organoids using a miniaturized spinning bioreactor. *Nat. Protoc.* 13 565–580. 10.1038/nprot.2017.152 29470464 PMC6241211

[B48] Qian X. Nguyen H. N. Song M. M. Hadiono C. Ogden S. C. Hammack C. (2016). Brain-region-specific organoids using mini-bioreactors for modeling ZIKV exposure. *Cell* 165 1238–1254. 10.1016/j.cell.2016.04.032 27118425 PMC4900885

[B49] Qian X. Song H. Ming G. L. (2019). Brain organoids: advances, applications and challenges. *Development* 146:dev166074 10.1242/dev.166074 PMC6503989 30992274

[B50] Quadrato G. Nguyen T. Macosko E. Z. Sherwood J. L. Yang S. M. Berger D. R. (2017). Cell diversity and network dynamics in photosensitive human brain organoids. *Nature* 545 48–53. 10.1038/nature22047 28445462 PMC5659341

[B51] Renner M. Lancaster M. A. Bian S. Choi H. Ku T. Peer A. (2017). Self-organized developmental patterning and differentiation in cerebral organoids. *EMBO J.* 36 1316–1329. 10.15252/embj.201694700 28283582 PMC5430225

[B52] Reynolds B. A. Rietze R. L. (2005). Neural stem cells and neurospheres – Re-evaluating the relationship. *Nat. Methods* 2 333–336. 10.1038/nmeth758 15846359

[B53] Reynolds B. A. Weiss S. (1992). Generation of neurons and astrocytes from isolated cells of the adult mammalian central nervous system. *Science* 255 1707–1710. 10.1126/science.1553558 1553558

[B54] Robertson F. L. Marqués-Torrejón M. A. Morrison G. M. Pollard S. M. (2019). Experimental models and tools to tackle glioblastoma. *DMM Dis. Model. Mech.* 12:dmm040386 10.1242/dmm.040386 PMC6765190 31519690

[B55] Sachs N. de Ligt J. Kopper O. Gogola E. Bounova G. Weeber F. (2018). A living biobank of breast cancer organoids captures disease heterogeneity. *Cell* 172 373–386.e10. 10.1016/j.cell.2017.11.010 29224780

[B56] Schüller U. Heine V. M. Mao J. Kho A. T. Dillon A. K. Han Y. G. (2008). Acquisition of granule neuron precursor identity is a critical determinant of progenitor cell competence to form shh-induced medulloblastoma. *Cancer Cell* 14 123–134. 10.1016/j.ccr.2008.07.005 18691547 PMC2597270

[B57] Singh S. K. Hawkins C. Clarke I. D. Squire J. A. Bayani J. Hide T. (2004). Identification of human brain tumour initiating cells. *Nature* 432 396–401. 10.1038/nature03128 15549107

[B58] Svendsen C. N. ter Borg M. G. Armstrong R. J. Rosser A. E. Chandran S. Ostenfeld T. (1998). A new method for the rapid and long term growth of human neural precursor cells. *J. Neurosci. Methods* 85 141–152. 10.1016/s0165-0270(98)00126-5 9874150

[B59] Swartling F. J. Èanèer M. Frantz A. Weishaupt H. Persson A. I. (2015). Deregulated proliferation and differentiation in brain tumors. *Cell Tissue Res.* 359 225–254. 10.1007/s00441-014-2046-y 25416506 PMC4286433

[B60] Tirosh I. Venteicher A. S. Hebert C. Escalante L. E. Patel A. P. Yizhak K. (2016). Single-cell RNA-seq supports a developmental hierarchy in human oligodendroglioma. *Nature* 539 309–313. 10.1038/nature20123 27806376 PMC5465819

[B61] Tunici P. Bissola L. Lualdi E. Pollo B. Cajola L. Broggi G. (2004). Genetic alterations and in vivo tumorigenicity of neurospheres derived from an adult glioblastoma. *Mol. Cancer* 3:25 10.1186/1476-4598-3-25 PMC524518 15469606

[B62] Tuveson D. Clevers H. (2019). Cancer modeling meets human organoid technology. *Science (80-)* 364 952–955. 10.1126/science.aaw6985 31171691

[B63] Van De Wetering M. Francies H. E. Francis J. M. Bounova G. Iorio F. Pronk A. (2015). Prospective derivation of a living organoid biobank of colorectal cancer patients. *Cell* 161 933–945. 10.1016/j.cell.2015.03.053 25957691 PMC6428276

[B64] van Pel D. M. Harada K. Song D. Naus C. C. Sin W. C. (2018). Modelling glioma invasion using 3D bioprinting and scaffold-free 3D culture. *J. Cell Commun. Signal.* 12 723–730. 10.1007/s12079-018-0469-z 29909492 PMC6235776

[B65] Vanner R. J. Remke M. Gallo M. Selvadurai H. J. Coutinho F. Lee L. (2014). Quiescent Sox2+ cells drive hierarchical growth and relapse in sonic hedgehog subgroup medulloblastoma. *Cancer Cell* 26 33–47. 10.1016/j.ccr.2014.05.005 24954133 PMC4441014

[B66] Velasco S. Kedaigle A. J. Simmons S. K. Nash A. Rocha M. Quadrato G. (2019). Individual brain organoids reproducibly form cell diversity of the human cerebral cortex. *Nature* 570 523–527. 10.1038/s41586-019-1289-x 31168097 PMC6906116

[B67] Vukicevic V. Jauch A. Dinger T. C. Gebauer L. Hornich V. Bornstein S. R. (2010). Genetic instability and diminished differentiation capacity in long-term cultured mouse neurosphere cells. *Mech. Ageing Dev.* 131 124–132. 10.1016/j.mad.2010.01.001 20074583

[B68] Weller M. Roth P. Preusser M. Wick W. Reardon D. A. Platten M. (2017). Vaccine-based immunotherapeutic approaches to gliomas and beyond. *Nat. Rev. Neurol.* 13 363–374. 10.1038/nrneurol.2017.64 28497804

[B69] Yan H. H. N. Siu H. C. Law S. Ho S. L. Yue S. S. K. Tsui W. Y. (2018). A comprehensive human gastric cancer organoid biobank captures tumor subtype heterogeneity and enables therapeutic screening. *Cell Stem Cell* 23 882–897.e11. 10.1016/j.stem.2018.09.016 30344100

[B70] Yi H. G. Jeong Y. H. Kim Y. Choi Y. J. Moon H. E. Park S. H. (2019). A bioprinted human-glioblastoma-on-a-chip for the identification of patient-specific responses to chemoradiotherapy. *Nat. Biomed. Eng.* 3 509–519. 10.1038/s41551-019-0363-x 31148598

[B71] Yuan X. Curtin J. Xiong Y. Liu G. Waschsmann-Hogiu S. Farkas D. L. (2004). Isolation of cancer stem cells from adult glioblastoma multiforme. *Oncogene* 23 9392–9400. 10.1038/sj.onc.1208311 15558011

[B72] Zanders E. D. Svensson F. Bailey D. S. (2019). Therapy for glioblastoma: is it working? *Drug Discov. Today* 24 1193–1201. 10.1016/j.drudis.2019.03.008 30878561

